# Quality of Life in Adults With Severe Obesity: A Systematic Review and Meta‐Analysis of Cross‐Sectional Studies

**DOI:** 10.1155/jobe/6951531

**Published:** 2026-10-17

**Authors:** Tatiane Salgado Galvão de Macedo, Marina Magno do Nascimento Pereira, Thamíris do Amaral Roxo Pereira, Ana Clara Rodrigues de Assumpção, Flávia dos Santos Barbosa Brito, Cintia Chaves Curioni

**Affiliations:** ^1^ Postgraduate Program in Food Nutrition and Health Institute of Nutrition State University of Rio de Janeiro São Francisco Xavier Street, 524 Rio de Janeiro Rio de Janeiro Brazil uerj.br; ^2^ Department of Social Nutrition Institute of Nutrition State University of Rio de Janeiro São Francisco Xavier Street, 524 Rio de Janeiro Rio de Janeiro Brazil uerj.br

**Keywords:** physical and mental health, quality of life, severe obesity, systematic review

## Abstract

**Purpose:**

This systematic review aimed to synthesize evidence on the association between severe obesity (body mass index [BMI] ≥ 40 kg/m^2^) and quality of life (QoL) in adults who had not undergone bariatric surgery or other interventions specifically targeting QoL, and to identify the QoL domains in which lower scores were most consistently observed.

**Methods:**

Cohort and cross‐sectional studies published up to March 2024 were eligible. The review protocol was registered in PROSPERO (CRD42023374612). PubMed, Embase, Scopus, Web of Science, SciELO, CINAHL, and LILACS were searched. Two independent reviewers selected studies, extracted data, and assessed methodological quality using the National Heart, Lung, and Blood Institute tool. Findings were synthesized narratively and through an exploratory random‐effects meta‐analysis of absolute SF‐36 mean scores.

**Results:**

Twenty cross‐sectional studies were included. The SF‐36 and its variations were the most frequently used instruments. Across studies with BMI comparison groups, higher BMI categories were generally associated with lower QoL scores. The lowest scores were most consistently observed for physical functioning, role due to physical health, bodily pain, and general health. Among the mental health‐related domains, vitality showed the most consistently reduced scores. Between‐study heterogeneity was high for most pooled estimates.

**Conclusion:**

Across the available cross‐sectional evidence, adults with severe obesity generally had lower QoL, particularly in physical domains. However, substantial heterogeneity and the absence of longitudinal evidence limit certainty and preclude causal interpretation. Longitudinal studies using standardized QoL measures are needed.

## 1. Introduction

Obesity is a chronic noncommunicable disease (NCD) and a major global public health challenge associated with metabolic, cardiovascular, pulmonary, hepatic, musculoskeletal, and oncologic conditions [1–3]. Severe obesity, defined as a body mass index (BMI) ≥ 40 kg/m^2^, is associated with increased physical disability, psychological morbidity, and premature mortality [4–7]. Its increasing prevalence across countries at all income levels underscores the importance of examining health outcomes that extend beyond morbidity and mortality [8, 9].

Quality of life (QoL) is defined by the World Health Organization as the perception individuals have of their position in life within the context of their culture and value systems and in relation to their goals, expectations, standards, and concerns [10]. This multidimensional construct encompasses physical health, emotional well‐being, interpersonal relationships, and social context [11, 12].

Among adults with severe obesity, lower QoL scores have been reported in association with limitations in daily activities and mobility, pain, emotional difficulties, and challenges in interpersonal and occupational functioning [13–15]. The relationship between obesity and mental health is complex and potentially bidirectional: psychiatric conditions and some treatments may contribute to weight gain, while weight stigma may intensify psychological distress [14, 16].

Research indicates an association between higher BMI and greater impairment in QoL [13, 17, 18]. A previous meta‐analysis examined BMI as a continuous exposure and was restricted to the SF‐36 instrument rather than focusing specifically on adults with severe obesity [19]. Other systematic reviews addressing severe obesity and QoL have predominantly evaluated individuals undergoing bariatric surgery, including changes after surgery or comparisons between preoperative and postoperative QoL [20–25]. Consequently, evidence concerning adults with severe obesity who have not undergone surgery or other interventions specifically targeting QoL remains less clearly synthesized.

Therefore, this systematic review aimed to identify and synthesize evidence on the association between severe obesity and QoL among adults who had not undergone bariatric surgery or other interventions specifically targeting QoL, with particular attention to the QoL domains and subdomains in which lower scores were most consistently observed.

## 2. Methods

This systematic review was conducted and reported in accordance with the Preferred Reporting Items for Systematic Reviews and Meta‐Analyses (PRISMA) 2020 statement [26] to address the research question: “Which QoL domains show the lowest scores among adults with severe obesity who have not undergone bariatric surgery or other interventions?” The review protocol was registered in the PROSPERO database (CRD42023374612).

### 2.1. Search Strategy

Searches of PubMed, Embase, Scopus, Web of Science, SciELO, CINAHL, and LILACS were completed in March 2024 and identified eligible studies published up to that date. Search strategies combined search terms with Boolean operators and were adapted for each database. The complete search strategies for PubMed, Embase, Scopus, Web of Science, LILACS, SciELO, and CINAHL are provided in Supporting Tables S1, S2, S3, S4, S5, S6, and S7, respectively. Additionally, the reference lists of reviews and included studies were manually searched to identify additional eligible studies.

### 2.2. Eligibility Criteria

Inclusion criteria encompassed original scientific articles of cross‐sectional and cohort studies that met the following PECO criteria:

• *Population (P):* Adults (≥ 18 years). Studies including both adults and older adults were eligible.
• *Exposure (E):* Individuals with severe obesity (BMI ≥ 40 kg/m^2^).
• *Comparator (C):* Studies could include comparators based on different BMI levels (as categorized by the authors) or no comparator.
• *Outcome (O):* QoL and its domains, assessed using validated instruments (generic or obesity‐specific). Studies could report overall QoL and/or individual scores for each domain or subdomain.

No restrictions on date or language were applied. Exclusion criteria encompassed studies involving individuals who had undergone bariatric surgery or other interventions that could influence QoL outcomes, such as structured weight management programs including dietary re‐education, supervised physical exercise, behavioral therapy, or combinations of these approaches. Studies focusing on pregnant women or exclusively on older adults, unpublished studies, studies without full‐text access, or those lacking additional eligibility information from the authors upon request were also excluded.

### 2.3. Study Selection

Search results were transferred to the Rayyan QCRI platform for systematic review management [27]. One reviewer (T.S.G.M.) removed the duplicates, and the remaining records underwent screening independently by four reviewers, working in pairs (T.S.G.M. and C.C.C. or M.M.N.P. or T.A.R.P.). Each pair reviewed titles, abstracts, and full texts to determine study eligibility. In cases of disagreement, two reviewers conducted a further analysis and reached a consensus through discussion.

### 2.4. Data Extraction

Two reviewers (T.S.G.M. and C.C.C.) independently extracted the data from eligible studies using an online Google Forms tool. Discrepancies were resolved through a consensus discussion or by revisiting the study. The original authors were contacted via email for additional data if needed.

Extracted data included study characteristics (authors, study type, year of publication, location); participant characteristics (age, sex, population origin, comorbidities, sociodemographic features); study objectives; methods (study design, QoL measurement instruments, statistical methods); and results (sample size for cross‐sectional studies or cohort baselines and follow‐ups, BMI data, overall QoL scores, and domain/subdomain scores as reported by the authors).

### 2.5. Quality Assessment

Two reviewers (T.S.G.M. and C.C.C.) independently evaluated the methodological quality of the studies using the *Quality Assessment Tool for Observational Cohort and Cross-Sectional Studies* from the National Heart, Lung, and Blood Institute (NHLBI)—National Institutes of Health (NIH) [28]. This tool includes 14 items addressing the research question, study population, eligibility criteria, sample size, exposure and outcome measurements, follow‐up, follow‐up rate, and statistical analyses. Responses were classified as “Yes,” “No,” or “Other” (CD: cannot be determined; NA: not applicable; NR: not reported). For cross‐sectional studies, nine items were assessed, as items 6, 7, 10, 12, and 13 are specific to cohort studies. Disagreements were resolved through consensus.

### 2.6. Data Synthesis

The results were presented narratively, with study characteristics summarized in a table. Study selection was illustrated using a PRISMA flowchart. Due to heterogeneity among studies, results were grouped and presented according to the QoL measurement instrument used.

A meta‐analysis was conducted to synthesize evidence from studies that used the SF‐36 QoL instrument using the random‐effects REML (restricted maximum likelihood) model. The meta‐analysis examined the mean scores across studies for each SF‐36 domain (mental component summary [MCS] and physical component summary [PCS]) and subdomains (physical functioning, physical role, bodily pain, general health, mental health, emotional role, social functioning, and vitality) stratified by population (men and women, men only, or women only).

Heterogeneity was assessed visually using Forest plots and the I^2^ test, which quantifies inconsistency between studies. I^2^ values were categorized as low (25%–50%), moderate (50%–75%), or high (> 75%) [29]. Subgroup analyses were performed based on sex.

All statistical analyses were conducted using STATA Version 16.0 (StataCorp LLC, Texas, USA).

## 3. Results

The initial search identified 1784 records in the databases, with an additional 38 found through citation tracking of potentially eligible studies. After removing duplicates, 1419 publications remained. Screening the titles and abstracts excluded 1256 publications. Among the reports assessed at full text, two cohort studies were identified as potentially eligible; however, they were excluded because they did not meet the prespecified eligibility criteria, resulting in a final sample of 20 cross‐sectional studies included in this review [30–49] (Figure 1).

**FIGURE 1:**
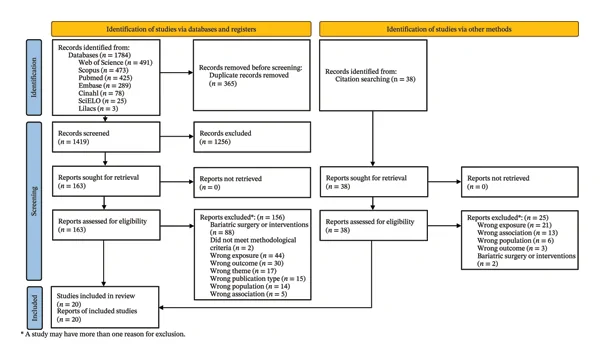
PRISMA flowchart of the study search and selection process.

### 3.1. Characteristics of Included Studies

All 20 studies included in the final analysis had a cross‐sectional design (Table 1). Most studies used a single instrument to measure QoL, with the SF‐36 or its variations (SF‐12, SF‐6D) being the most common, appearing in 12 studies [31, 33, 34, 36, 37, 40, 41, 43–46, 48]. Other instruments included the EQ‐5D (six studies) [30, 32, 39, 45, 47, 49], IWQOL‐Lite (one study) [38], WHOQOL‐Bref (one study) [42], and BRFSS HRQOL‐CDC (one study) [35] (Table 1). Detailed results from studies that used QoL instruments other than the SF‐36 are presented in the Supporting Information.

**TABLE 1:** Characteristics of the included studies.

Study/country	HRQoL instruments	Origin of the sample	Comparison groups	Sample total/obesity III (*n*)	Female total/obesity III (%)	BMI obesity III (kg/m^2^)^a^ mean (SD)	Age (years) total/obesity III mean (SD)
Apple et al./USA [30]	EQ‐5D	Mid‐South Clinical Data Research Network	No categories	10,133/1974	71.7%/76.6%	NR	52.1 (37.2–63.3)^c^/51.3 (40.1–60.5)^c^
Arbex et al./Brazil [31]	SF‐36	Outpatients of the Department of Nutrition	BMI categories	113/46	71.7%/69.6%	NR	48.9 (14.9)/43.2 (14.0)
Busutil et al./Spain [32]	EQ‐5D	Spanish National Health Survey (SNHS) 2011–2012	BMI categories	18,682/161	NR	NR	NR
Corica et al./Italy [33]	SF‐36	Quality of life in Obesity: Evaluation and Disease Surveillance study (QUOVADIS)	BMI categories (only obesity categories)	1822/604	77.9%/NR	NR	20–65^b^/NR
Doll et al./United Kingdom [34]	SF‐36	General practitioner records for four English countries	BMI categories	8889/69	56%/NR	NR	41.6 (12.6)/NR
Ford et al./USA [35]	BRFSS HRQOL ‐ CDC	Behavioral Risk Factor Surveillance System (BRFSS)	BMI categories	109,076/1598	56.9%/66%	NR	NR/44.6 (0.5)
Hopman et al./Canada [36]	SF‐36	Canadian Multicentre Osteoporosis Study (CaMos)	BMI categories	9094/136	69.3%/85.3%	NR	25–75+^b^/NR
Knoesen et al./Australia [37]	SF‐36	Obesity Have Your Say study	BMI categories (only obesity categories)	118/46	77.9%/89.1%	46.99 (6.42)	45.3 (11.8)/44.5 (10.3)
Kolotkin et al./USA [38]	IWQOL‐lite	Community Volunteers recruited from general population	BMI categories	425/NR	45.9%/NR	NR	NR/F: 41.3 (13.7); M: 38.1 (11.0)
Luah et al./England [39]	EQ‐5D	Health Survey of England (HSE)	BMI categories	12,158/466	51%/72.1%	NR	18–75+^b^/NR
Palmeira et al./Brazil [40]	SF‐36	Reference outpatient clinic for obesity	BMI categories	82/26	100%/100%	NR	48.6 (8.6)/NR
Pazzianotto‐Forti et al./Brazil [41]	SF‐36	Volunteered from the community or the obesity treatment clinic	BMI categories	133/44	100%/100%	NR	NR/41.3 (11.4)
Pimenta et al./Brazil [42]	WHOQOL‐Bref	Service of Applied Psychology	BMI categories	60/20	50%/50%	NR	20–60^b^/NR
Renzaho et al./Australia [43]	SF‐36	HILDA survey	BMI categories	9728/178	49.9%/69.1%	NR	F: 49.1 (16.8); M: 47.7 (16.4)/NR
Rozjabek et al./USA [44]	SF‐36	National Health and Wellness Survey (NHWS) 2018	BMI categories	69,742/4590	NR/60.2%	NR	NR/46.9^d^
Sach et al./United Kingdom [45]	SF‐6D, EQ‐5D, EQ‐VAS	UK general practice	BMI categories	1730/16	55.3%/68.8%	NR	64.7^d^; 45.9–99.7^b^/NR
Slagter et al./Netherlands [46]	SF‐36	Dutch Lifelines Cohort Study	BMI categories	13,686/835	62%/80.1%	NR	F: 47 (12); M: 48 (11)/NR
Soltoft et al./England [47]	EQ‐5D	HSE survey	BMI categories	14,416/296	55.5%/NR	NR	F: 49.4 (18.3); M: 48.8 (17.5)/NR
Stephenson et al./United Kingdom [49]	EQ‐5D	Yorkshire Health Study	BMI categories	64,631/1678	63.1%/76.1%	NR	50.4 (17.7)/49.6 (14.9)
Yancy et al./USA [48]	SF‐36	Outpatients from the primary care	BMI categories	1168/30	0	NR	54.7 (5.6)/54.8 (5.6)

*Note:* BRFSS HRQOL‐CDC, Behavioral Risk Factor Surveillance System Health‐Related Quality of Life—Centers for Disease; EQ‐5D, EuroQoL 5 dimension; EQ‐VAS, EuroQoL visual analogue scale; F, female; IWQOL‐lite, Impact of Weight on Quality of Life‐Lite; M, male; SF‐12, 12‐Item Short Form Health Survey; SF‐36, 36‐Item Short Form Health Survey; SF‐6D, Short‐Form 6 Dimensions; WHOQOL‐Bref, World Health Organization Quality of Life Scale.

Abbreviations: BMI, body mass index; NA, not applicable; NR, not reported; SD, standard deviation.

^a^Studies that included multiple categories did not specify the mean BMI for the group of severe obesity and were therefore classified as not reported (NR).

^b^Age range.

^c^Age presented in median (interquartile range).

^d^Age presented in mean.

Regarding comparators used in the studies, most compared BMI categories. Only one study did not use comparison categories [30], while two others compared only obesity categories [33, 37] (Table 1).

The geographic distribution of the studies was primarily in the Americas (10 studies) [30, 31, 35, 36, 38, 40–42, 44, 48], followed by Europe (eight studies) [32–34, 39, 45–47, 49] and Oceania (two studies) [37, 43] (Table 1). The studies were published between 2000 and 2024, with significant variation in the sample sizes of participants with severe obesity. Only four studies had samples larger than 1000 participants [30, 35, 44, 49]. One study did not report the specific number of participants with severe obesity in its sample [38] (Table 1).

The individual results of the included studies, organized by BMI categories, are available in the Supporting Information. Overall, most studies reported an inverse association between BMI and QoL, indicating that higher BMI values were associated with lower QoL. It should be noted that the tables presented in this review refer specifically to individuals with severe obesity.

Table 2 and Figures 2 and 3 summarize the findings of studies that evaluated QoL in individuals with severe obesity using the SF‐36. This widely used instrument enabled robust comparative analysis. The data were segmented by sex and general sample, according to the categorization in the original studies. Lower SF‐36 scores indicate worse QoL. The studies by Palmeira [40] and Pazzianato‐Forti [41] present some data in medians, which explains the absence of some data in Figure 2.

**TABLE 2:** Quality of life results in studies using the SF‐36 instrument for individuals with severe obesity—mean (SD).

Study	PCS	MCS	Physical function	Role physical	Bodily pain	General health	Mental health	Role emotional	Social function	Vitality
*TOTAL*										
Arbex et al. [31]	44.2 (4.1)	—	—	—	—	—	—	—	—	—
Doll et al. [34]	40.6 (13.1)	48.1 (10.9)	65.7 (25.8)	74.6 (26.8)	64.9 (25.4)	56.9 (24.2)	66.3 (20.1)	78.8 (26.0)	72.1 (29.0)	47.3 (22.7)
Knoesen et al. [37]	45.1 (19.5)	58.4 (21.7)	55.3 (26.8)	50.5 (40.3)	26.5 (12.5)	47.1 (12.7)	65.7 (20.9)	67.4 (39.4)	60.9 (26.3)	40.3 (19.4)
Palmeira et al. [40]	—	—	45 (25–80)	25 (0–100)	40 (20–61)	48 (30–67)	56 (40–80)	33 (0–100)	62 (50–75)	50 (20–80)
Pazzianotto‐Forti et al. [41]	—	—	50 (35–67)	50 (0–100)	51 (30–62)	53.7 (15.1)	68 (44–80)	66 (33–100)	69.3 (28.9)	60 (35–70)
Rozjabek et al. [44]	43	43.2	42.5	43.1	43.4	42	43.6	42	42.7	43.1
Yancy et al. [48]	28.5 (0.2)	48.8 (15.4)	35.0 (23.1)	27.5 (37.3)	38.8 (25.1)	47.2 (23.6)	67.5 (28.5)	68.9 (42.8)	57.9 (29.8)	33.0 (27.1)

*MALE*										
Hopman et al. [36]	40.55 (10.0)	56.0 (6.3)	63.0 (27.4)	62.5 (41.7)	62.8 (24.2)	66.3 (19.0)	84.0 (9.3)	81.7 (35.0)	80.0 (21.2)	65.3 (16.4)
Renzaho et al. [43]	42.9 (12.3)	49.9 (11.5)	66.3 (30.4)	61.0 (44.1)	59.2 (30.6)	51.2 (23.1)	70.9 (21.6)	73.8 (41.7)	74.1 (29.6)	57.7 (23.7)

*FEMALE*										
Hopman et al. [36]	38.3 (12.5)	52.0 (9.9)	55.3 (28.4)	53.9 (42.3)	57.7 (25.9)	60.2 (24.5)	75.4 (17.7)	72.1 (39.6)	76.0 (23.7)	53.8 (20.7)
Renzaho et al. [43]	42.6 (11.6)	47.9 (11.3)	63.1 (26.6)	60.1 (43.4)	56.4 (27.3)	53.1 (23.2)	67.6 (19.5)	72.5 (40.5)	71.1 (26.9)	47.7 (22.3)

*Note:* SF‐36, 36‐Item Short Form Health Survey.

Abbreviations: MCS, mental component score; PCS, physical component score; SD, standard deviation.

**FIGURE 2:**
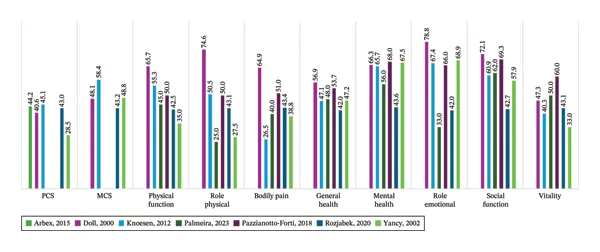
SF‐36 results by domains and subdomains for individuals with severe obesity for total (male and female) population—mean. Legend: MCS, mental component score; PCS, physical component score; SD, standard deviation; SF‐36, 36‐Item Short Form Health Survey.

**FIGURE 3:**
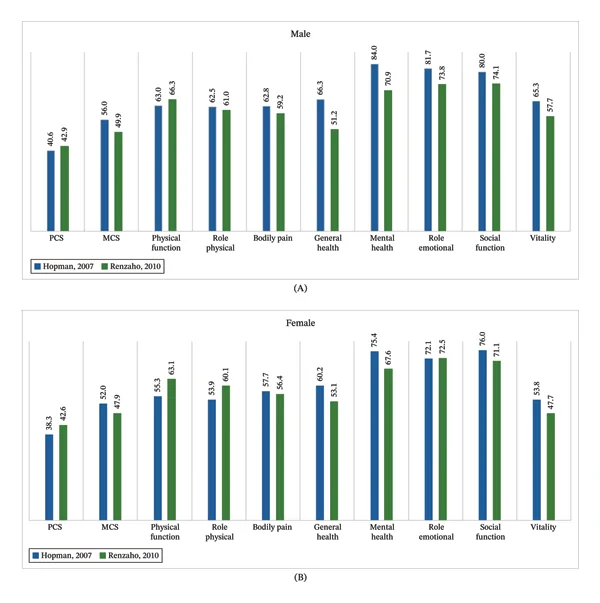
SF‐36 results by domains and subdomains for individuals with severe obesity by sex—mean. MCS, mental component score; PCS, physical component score; SD, standard deviation; SF‐36, 36‐Item Short Form Health Survey.

In studies evaluating the general sample [31, 34, 37, 41, 44, 48], mean PCS ranged from 28.5 to 45.1, while MCS ranged from 43.2 to 58.4. Domains related to physical health, such as physical functioning, role‐physical, bodily pain, and general health, showed the lowest values, with the lowest scores being 35.0, 27.5, 26.5, and 42.0, respectively. An exception was the study by Doll [34], which reported high scores in the domains of physical functioning, role‐physical, and bodily pain, with values of 65.7, 74.6, and 64.9, respectively (Figure 2).

In the mental health domains, scores were generally high, exceeding 57.9 [48], except for the vitality domain, which showed reduced scores, with the lowest being 33.0 [48]. The study by Rozjabek [44] exhibited significantly low values in the mental health, emotional role, and social functioning domains, with scores of 43.6, 42.0, and 42.7, respectively, diverging from the other studies (Figure 2).

In studies that evaluated by sex [36, 43], both men and women showed lower PCS scores compared to MCS, maintaining the pattern of physical domains scoring lower than mental domains, except for vitality (Figure 3A,B).

Two studies using the SF‐36 were excluded from the synthesis table and meta‐analysis because their results were not directly comparable with those of the other studies; their findings are presented in Supporting Table S10 [33, 46].

The studies by Busutil et al. [32] and Søltoft et al. [47], using the EQ‐5D instrument, reported the highest proportions of problems in the mobility and pain domains among individuals with severe obesity. Busutil et al. [32] found rates of 44.7% for mobility and 47.2% for pain. Similarly, Søltoft et al. [47] observed high frequencies in these domains, particularly among women, who reported greater difficulties than men (Supporting Tables S8 and S11).

Results from studies using other QoL instruments are presented in Supporting Tables S9 and S12.

### 3.2. Meta‐Analysis

A meta‐analysis was conducted to synthesize PCS and MCS scores among adults with severe obesity using the SF‐36. The pooled mean scores were estimated for the overall study population and for sex‐specific samples.

The pooled PCS score was numerically lower in the female‐only and mixed‐sex analyses than in the male‐only analysis, indicating lower reported physical health‐related quality‐of‐life scores in these analyses. Substantial heterogeneity was observed in the female‐only and mixed‐sex analyses, indicating considerable between‐study variability that was not explained by sex composition alone (Figure 4).

**FIGURE 4:**
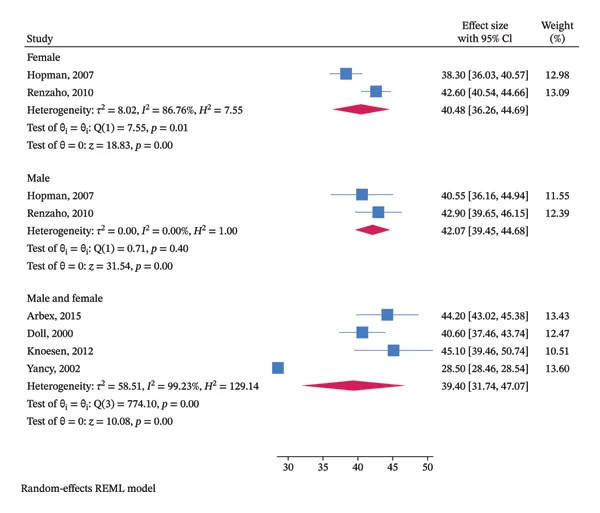
SF‐36 physical component score (PCS) meta‐analysis. 95% CI, 95% confidence interval; SF‐36, 36‐Item Short Form Health Survey.

The pooled MCS score was numerically lower in the female‐only analysis than in the male‐only and mixed‐sex analyses, indicating lower reported mental health‐related quality‐of‐life scores in the female‐only analysis. Substantial heterogeneity was also observed across all population strata, indicating considerable between‐study variability that was not explained by sex composition alone (Figure 5).

**FIGURE 5:**
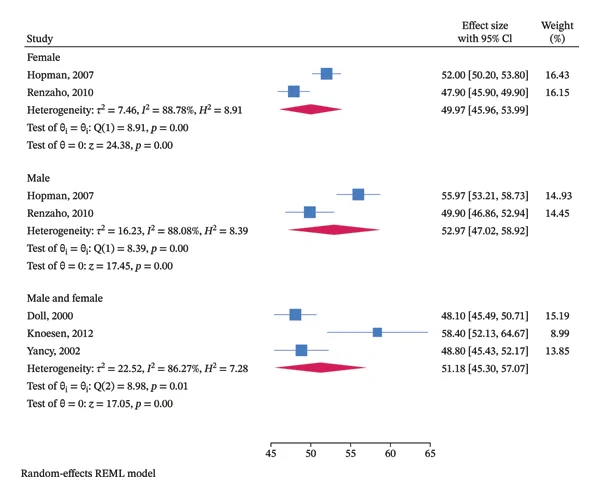
SF‐36 mental component score (MCS) meta‐analysis. 95% CI, 95% confidence interval; SF‐36, 36‐Item Short Form Health Survey.

Additionally, meta‐analyses were conducted for all SF‐36 QoL domains, including Physical Function, Role‐Physical, Bodily Pain, General Health, Mental Health, Role Emotional, Social Function, and Vitality. Detailed results are presented in Supporting Figures S1–S8, respectively.

### 3.3. Methodological Quality Assessment of Included Studies

All studies clearly defined the research question and outcome measures [30–49]. However, 16 studies did not meet the criteria related to sample size justification [30, 32–35, 37, 38, 40, 42–49]. Eleven studies used indirect measurements of obtaining exposure measurements [30–32, 34, 35, 37, 38, 42–45]; nine studies did not provide sufficient information, or it was unclear whether they met the participation rate criterion for eligible individuals [30, 36, 38, 41, 42, 44, 46, 48, 49]. Eight studies did not adequately address potential confounding variables [31, 33, 34, 38, 40–42, 44] (Table 3).

**TABLE 3:** Critical evaluation of studies included in the systematic review.

	Research question	Study population	Rate of eligible persons	Recruitment	Sample size justification	Exposure variability	Exposure measures	Outcome measures	Potential confounding variables
Apple et al. [30]	Y	Y	N	Y	N	Y	N	Y	Y
Arbex et al. [31]	Y	Y	Y	Y	Y	Y	CD	Y	N
Busutil et al. [32]	Y	Y	Y	Y	N	Y	N	Y	Y
Corica et al. [33]	Y	Y	Y	Y	N	Y	Y	Y	N
Doll et al. [34]	Y	N	Y	NR	N	Y	N	Y	N
Ford et al. [35]	Y	Y	Y	Y	N	Y	N	Y	Y
Hopman et al. [36]	Y	Y	N	Y	Y	Y	Y	Y	Y
Knoesen et al. [37]	Y	Y	Y	Y	N	Y	N	Y	Y
Kolotkin et al. [38]	Y	N	CD	N	N	Y	N	Y	N
Luah et al. [39]	Y	Y	Y	Y	Y	Y	Y	Y	Y
Palmeira et al. [40]	Y	Y	Y	Y	N	Y	Y	Y	N
Pazzianotto‐Forti et al. [41]	Y	N	CD	CD	Y	Y	Y	Y	N
Pimenta et al. [42]	Y	N	N	N	N	Y	N	Y	N
Renzaho et al. [43]	Y	Y	Y	Y	N	Y	N	Y	Y
Rozjabek et al. [44]	Y	CD	CD	CD	CD	Y	N	Y	N
Sach et al. [45]	Y	Y	Y	Y	N	Y	N	Y	Y
Slagter et al. [46]	Y	Y	CD	Y	N	Y	Y	Y	Y
Søltoft et al. [47]	Y	Y	Y	Y	N	Y	Y	Y	Y
Stephenson et al. [49]	Y	Y	CD	Y	N	N	Y	Y	Y
Yancy et al. [48]	Y	Y	N	Y	N	Y	Y	Y	Y
TOTAL (%):	100	75	55	75	20	95	45	100	60

*Note:* Y, yes; N, no.

Abbreviations: CD, cannot determine; NR, not reported.

## 4. Discussion

This review examined the association between severe obesity and QoL in adults who had not undergone interventions that could directly affect QoL, with particular attention to the domains showing the greatest impairment. Across the 20 included studies, QoL scores were generally lower in participants with higher BMI levels, with the lowest scores observed in adults with severe obesity. Lower scores were observed across several domains, although the magnitude and consistency of these differences varied among studies. These patterns may be related to the clinical, socioeconomic, and psychological characteristics frequently associated with severe obesity.

Despite the variability among studies, physical QoL domains generally showed greater impairment than mental domains. Lower scores were frequently observed for physical functioning, role‐physical, bodily pain, and general health, whereas vitality was among the mental domains with the lowest scores. These findings are consistent with previous systematic reviews examining associations between obesity, weight loss, and QoL [3, 18].

Severe obesity has been associated with mobility difficulties, fatigue, and chronic musculoskeletal pain, particularly in the knees and spine. These conditions may restrict daily activities such as walking, climbing stairs, dressing, and carrying objects. They may also be associated with reduced functional capacity, strength, and endurance and, consequently, with lower physical QoL [13, 50, 51]. Comorbidities such as T2DM, hypertension, sleep apnea, and cardiovascular diseases may contribute to the physical limitations reported among adults with severe obesity [2, 52–56].

Regarding mental QoL, obesity has been associated with depression, anxiety, and eating disorders. Weight stigma may contribute to psychological distress, which can be compounded by social and physical barriers to participation in everyday activities. Emotional coping strategies and social support may help preserve some aspects of mental QoL, even in the presence of substantial physical limitations [16, 57–59]. However, the instruments used in the included studies did not directly assess social support or coping strategies, limiting conclusions about their potential role.

Although mental component scores generally tended to be higher than physical component scores, lower vitality and difficulties related to energy, work, leisure activities, and self‐esteem were also reported [13, 18, 60, 61]. Physical and mental QoL are closely interrelated; however, the direction and temporal sequence of these relationships cannot be established from the available evidence. Therefore, assessment of both components may provide a more comprehensive understanding of the experiences of adults with severe obesity.

The findings also suggest that QoL assessment among adults with obesity may benefit from the combined use of generic and obesity‐specific instruments. Generic instruments facilitate comparisons across populations and health conditions but may be less sensitive to experiences specifically related to obesity. Obesity‐specific instruments, such as the IWQOL‐Lite, may complement generic measures by capturing aspects such as weight‐related physical limitations, self‐esteem, social interactions, and daily activities [12, 18].

From a clinical perspective, the observed patterns suggest that assessment of adults with severe obesity may consider dimensions beyond body weight, including mobility, chronic pain, physical and social limitations, psychological well‐being, stigma, and autonomy. These dimensions may help inform individualized and multidisciplinary care strategies that include psychological support, health‐promoting behaviors, pain management, and support for physical functioning. However, the effectiveness of these approaches cannot be determined from the studies included in this review [57, 62, 63].

The included studies showed variability in methodological quality, particularly regarding sampling strategies and the assessment or control of confounding variables. These limitations indicate the need for future studies with greater methodological rigor, including population‐based and longitudinal designs, to clarify the direction and magnitude of the observed associations.

Several limitations should be considered. Although a comprehensive search strategy was used, publication bias cannot be ruled out. In addition, the literature search was completed in March 2024; therefore, studies published or indexed after this date were not included. All included studies had a cross‐sectional design, which precludes establishing temporality or causal relationships between severe obesity and QoL. Substantial heterogeneity was observed in several meta‐analyses. Although random‐effects models using restricted maximum likelihood estimation were applied to account for expected between‐study variability, this approach does not eliminate the uncertainty associated with substantial heterogeneity. Differences in age, BMI severity, comorbidity profiles, recruitment settings, geographic context, sample composition, and SF‐36 administration or scoring procedures may have contributed to the observed variability. However, the limited number of studies precluded a more detailed investigation of these potential sources of heterogeneity.

The included studies also varied in methodological quality, particularly regarding sampling strategies and the assessment or control of confounding variables. Therefore, the pooled estimates should be interpreted as descriptive summaries of the SF‐36 scores reported in the included studies rather than as population‐representative reference values. These factors reduced comparability and limited the number of studies that could be included in the meta‐analysis.

Nevertheless, this review provides a focused synthesis of the association between severe obesity and QoL in adults who had not undergone bariatric surgery or other interventions that could directly affect QoL. This focus is clinically relevant because not all eligible adults undergo surgery, and access to newer pharmacological treatments may be limited by cost, availability, or coverage. By characterizing QoL in this population, the findings support a more comprehensive, person‐centered assessment that extends clinical attention beyond body weight alone.

The inclusion of studies using validated QoL instruments and the separate synthesis of physical and mental health‐related QoL components are strengths. Further population‐based and longitudinal research using standardized definitions, appropriate comparison groups, and instruments suitable for adults with severe obesity is needed to provide more robust evidence.

## 5. Conclusion

This systematic review found that, among adults with severe obesity who had not undergone bariatric surgery or other QoL‐targeted interventions, higher BMI categories were generally associated with lower QoL scores, particularly in physical functioning, role‐physical, bodily pain, and general health. Vitality was the mental domain in which lower scores were most consistently observed. However, the magnitude and consistency of these associations varied across studies. Further population‐based and longitudinal studies using standardized QoL measures and appropriate control of confounding factors are needed to clarify these associations.

## Author Contributions

Review conception and writing of the protocol: Tatiane Salgado Galvão de Macedo, Cintia Chaves Curioni, Flávia dos Santos Barbosa Brito; Literature search: Tatiane Salgado Galvão de Macedo; Study Selection: Tatiane Salgado Galvão de Macedo, Cintia Chaves Curioni, Marina Magno do Nascimento Pereira, Thamíris do Amaral Roxo Pereira; Data Extraction and Assessment of methodological quality: Tatiane Salgado Galvão de Macedo, Cintia Chaves Curioni; Data Analysis: Tatiane Salgado Galvão de Macedo, Cintia Chaves Curioni; Writing–original draft preparation: Tatiane Salgado Galvão de Macedo; Writing–review and editing: Tatiane Salgado Galvão de Macedo, Cintia Chaves Curioni, Flávia dos Santos Barbosa Brito, Marina Magno do Nascimento Pereira, Thamíris do Amaral Roxo Pereira, Ana Clara Rodrigues de Assumpção; Technical review: Cintia Chaves Curioni, Flávia dos Santos Barbosa Brito; Supervision: Tatiane Salgado Galvão de Macedo, Cintia Chaves Curioni. The first draft of the manuscript was written by Tatiane Salgado Galvão de Macedo, and all authors commented on previous versions of the manuscript.

## Funding

The main author, Tatiane Salgado Galvão de Macedo, was supported by the Carlos Chagas Filho Foundation for Research Support in the State of Rio de Janeiro (FAPERJ), Brazil, through a doctoral scholarship (Request/Process E‐26/201.516/2021 (266023)).

## Disclosure

The funding institution had no involvement in the design of the protocol, analysis plan, data collection, or analysis. Additionally, this organization did not contribute to the interpretation or publication of the results. All authors read and approved the final manuscript.

## Ethics Statement

The authors have nothing to report.

## Consent

The authors have nothing to report.

## Conflicts of Interest

The authors declare no conflicts of interest.

## Supporting Information

Additional supporting information can be found online in the Supporting Information section.

## Supporting information


**Supporting Information** The following supporting information is available through the online version of this article at the publisher’s website: Supporting Table S1: Search strategy used in MEDLINE via PubMed. Supporting Table S2: Search strategy used in Embase. Supporting Table S3: Search strategy used in Scopus. Supporting Table S4: Search strategy used in Web of Science. Supporting Table S5: Search strategy used in LILACS via BVS. Supporting Table S6: Search strategy used in SciELO. Supporting Table S7: Search strategy used in CINAHL via EBSCO. Supporting Table S8: Quality of Life results in studies using the EQ‐5D instrument among individuals with severe obesity—mean (SD). Supporting Table S9: Quality of Life results in studies using other instruments among individuals with severe obesity—mean (SD). Supporting Table S10: Quality of Life results from studies using the SF‐36 instrument by BMI categories. Supporting Table S11: Quality of Life results from studies using the EQ‐5D instrument by BMI categories. Supporting Table S12: Quality of Life results from studies using other instruments by BMI categories. Supporting Figure S1: Meta‐analysis of the SF‐36 Physical Function domain. Supporting Figure S2: Meta‐analysis of the SF‐36 Role Physical domain. Supporting Figure S3: Meta‐analysis of the SF‐36 Bodily Pain domain. Supporting Figure S4: Meta‐analysis of the SF‐36 General Health domain. Supporting Figure S5: Meta‐analysis of the SF‐36 Mental Health domain. Supporting Figure S6: Meta‐analysis of the SF‐36 Role Emotional domain. Supporting Figure S7: Meta‐analysis of the SF‐36 Social Function domain. Supporting Figure S8: Meta‐analysis of the SF‐36 Vitality domain.

## Data Availability

The datasets used and/or analyzed during the current review are available from the corresponding author upon reasonable request.
