# Supplementary material for: Quality of Life in Adults With Severe Obesity: A Systematic Review and Meta‐Analysis of Cross‐Sectional Studies
Source: J Obes. 2026 Oct 17;2026:6951531. doi: 10.1155/jobe/6951531 (PMC13676160; doi:10.1155/jobe/6951531)
Supplement: Supplementary file 1 [file JOBE-2026-6951531-s001.docx]

**Quality of Life in Adults with Severe Obesity: A Systematic Review and Meta-Analysis of Cross-Sectional Studies**

Tatiane Salgado Galvão de Macedo^1*^, Marina Magno do Nascimento Pereira^2^, Thamíris do Amaral Roxo Pereira^3^, Ana Clara Rodrigues de Assumpção^4^, Flávia dos Santos Barbosa Brito^5^ and Cintia Chaves Curioni^6^

^1^ Postgraduate Program in Food, Nutrition and Health, Institute of Nutrition, State University of Rio de Janeiro São Francisco Xavier Street, 524, Rio de Janeiro – RJ, Brazil; *****Corresponding Author: e-mail: [tatiane_galvao@outlook.com](mailto:tatiane_galvao@outlook.com)

^2^ Postgraduate Program in Food, Nutrition and Health, Institute of Nutrition, State University of Rio de Janeiro São Francisco Xavier Street, 524, Rio de Janeiro – RJ, Brazil; [marinamagno02@gmail.com](mailto:marinamagno02@gmail.com)

^3^ Postgraduate Program in Food, Nutrition and Health, Institute of Nutrition, State University of Rio de Janeiro, São Francisco Xavier Street, 524, Rio de Janeiro – RJ, Brazil; [thamirisroxo@gmail.com](mailto:thamirisroxo@gmail.com)

^4^ Postgraduate Program in Food, Nutrition and Health, Institute of Nutrition, State University of Rio de Janeiro, São Francisco Xavier Street, 524, Rio de Janeiro – RJ, Brazil; [anaclaranutricionista2@gmail.com](mailto:anaclaranutricionista2@gmail.com)

^5^ Department of Social Nutrition, Institute of Nutrition, State University of Rio de Janeiro, São Francisco Xavier Street, 524, Rio de Janeiro – RJ, Brazil; [barbosaflavia@bol.com.br](mailto:barbosaflavia@bol.com.br)

^6^ Department of Social Nutrition, Institute of Nutrition, State University of Rio de Janeiro, São Francisco Xavier Street, 524, Rio de Janeiro – RJ, Brazil; [cintiacurioni@gmail.com](mailto:cintiacurioni@gmail.com)

**SUPPLEMENTARY MATERIAL**

**Supplementary Table S1 –** Search strategy used in Medline via Pubmed

| Severe Obesity | Terms: MeSH and text word searches | "obesity, morbid"[MeSH Terms] OR "morbid obes*"[Text Word] OR "severe obes*"[Text Word] OR "Obesity class III"[Text Word] OR "class iii obes*"[Text Word] OR "severely obes*"[Text Word] |
| --- | --- | --- |
|  |  | **AND** |
| Quality of Life | Terms: MeSH and searches in titles and abstracts | "Quality of Life"[MeSH Terms] OR "Quality of Life"[Title/Abstract] OR "QOL"[Title/Abstract] OR "HRQOL"[Title/Abstract] OR "Life Quality"[Title/Abstract] OR "health-related quality of life"[Title/Abstract] OR "health-related quality of life"[Title/Abstract] |
|  |  | **AND** |
| Observational studies | Terms: MeSH and searches in titles and abstracts | "Cohort Studies"[MeSH Terms] OR "Cross-Sectional Studies"[MeSH Terms] OR "Epidemiologic Studies"[MeSH Terms] OR "epidemiologic stud*"[Title/Abstract] OR "cohort stud*"[Title/Abstract] OR "cohort analys*"[Title/Abstract] OR "cross-sectional"[Title/Abstract] OR "cross-sectional"[Title/Abstract] OR "observational stud*"[Title/Abstract] OR "prevalence stud*"[Title/Abstract] OR "incidence stud*"[Title/Abstract] OR "follow-up"[Title/Abstract] OR "followup"[Title/Abstract] OR "follow-up"[Title/Abstract] OR "longitudinal"[Title/Abstract] OR "prospective"[Title/Abstract] OR "retrospective"[Title/Abstract] |
|  |  | **NOT** |
| Limits for exclusion | Terms: MeSH | "Bariatric Surgery"[MeSH Terms] |
| Results: 425 | | |

**Supplementary Table S2 –** Search strategy used in Embase

| Severe Obesity | Emtree term - exploded and All Fields searches | 'morbid obesity'/exp OR 'morbid obesity' OR 'morbid obes*' OR 'severe obes*' OR 'obesity class iii' OR 'class iii obes*' OR 'severely obes*' |
| --- | --- | --- |
|  |  | **AND** |
| Quality of Life | Emtree term - exploded and abstract and title searches | 'quality of life'/exp OR 'quality of life':ab,ti OR qol:ab,ti OR hrqol:ab,ti OR 'life quality':ab,ti OR 'health-related quality of life':ab,ti |
|  |  | **AND** |
| Observational studies | Emtree term - exploded and abstract and title searches | 'cohort analysis'/exp OR 'cross-sectional study'/exp OR 'epidemiology'/exp OR 'epidemiologic stud*':ab,ti OR 'cohort stud*':ab,ti OR 'cohort analys*':ab,ti OR 'cross-sectional':ab,ti OR 'observational stud*':ab,ti OR 'prevalence stud*':ab,ti OR 'incidence stud*':ab,ti OR 'followup':ab,ti OR 'follow-up':ab,ti OR 'longitudinal':ab,ti OR 'prospective':ab,ti OR 'retrospective':ab,ti |
|  |  | **NOT** |
| Limits for exclusion | Emtree term - exploded | 'bariatric surgery'/exp |
| Results: 289 | | |

**Supplementary Table S3 –** Search strategy used in Scopus

| Severe Obesity | Searches in all fields | ( ALL ( "morbid obes*" ) OR ALL ( "severe obes*" ) OR ALL ( "obesity class iii" ) OR ALL ( "class iii obes*" ) OR ALL ( "severely obes*" ) ) |
| --- | --- | --- |
|  |  | **AND** |
| Quality of Life | Searches in titles, abstracts and keywords | ( TITLE-ABS-KEY ( "Quality of Life" ) OR TITLE-ABS-KEY ( qol ) OR TITLE-ABS-KEY ( hrqol ) OR TITLE-ABS-KEY ( "life quality" ) OR TITLE-ABS-KEY ( "health-related quality of life" ) OR TITLE-ABS-KEY ( "health related quality of life" ) ) |
|  |  | **AND** |
| Observational studies | Searches in titles, abstracts and keywords | ( TITLE-ABS-KEY ( "epidemiologic stud*" ) OR TITLE-ABS-KEY ( "cohort stud*" ) OR TITLE-ABS-KEY ( "cohort analys*" ) OR TITLE-ABS-KEY ( "cross-sectional" ) OR TITLE-ABS-KEY ( "observational stud*" ) OR TITLE-ABS-KEY ( "prevalence stud*" ) OR TITLE-ABS-KEY ( "incidence stud*" ) OR TITLE-ABS-KEY ( "followup" ) OR TITLE-ABS-KEY ( "follow-up" ) OR TITLE-ABS-KEY ( "longitudinal" ) OR TITLE-ABS-KEY ( "prospective" ) OR TITLE-ABS-KEY ( "retrospective" ) ) |
|  |  | **NOT** |
| Limits for exclusion | Searches in titles | ( TITLE ( "bariatric*" ) OR TITLE ( "metabolic surgery" ) OR TITLE ( "gastrectomy" ) OR TITLE ( "roux-en-y" ) OR TITLE ( "gastric bypass" ) OR TITLE ( "gastric balloon" ) ) |
| Results: 473 | | |

**Supplementary Table S4 –** Search strategy used in Web of Science

| Severe Obesity | Searches in all fields | "morbid obes*" or "severe obes*" or "obesity class iii" or "class iii obes*" or "severely obes*" |
| --- | --- | --- |
|  |  | **AND** |
| Quality of Life | Searches in Topic: Search titles, abstracts, author keywords and Keywords Plus. | "Quality of Life" OR qol OR hrqol OR "life quality" OR "health-related quality of life" OR "health related quality of life" |
|  |  | **AND** |
| Observational studies | Searches in Topic: Search titles, abstracts, author keywords and Keywords Plus. | "epidemiologic stud*" OR "cohort stud*" OR "cohort analys*" OR "cross-sectional" OR "observational stud*" OR "prevalence stud*" OR "incidence stud*" OR "followup" OR "follow-up" OR "longitudinal" OR "prospective" OR "retrospective" |
|  |  | **NOT** |
| Limits for exclusion | Searches in titles | "bariatric*" OR "metabolic surgery" OR "gastrectomy" OR "roux-en-y" OR "gastric bypass" OR "gastric balloon" |
| Results: 491 | | |

**Supplementary Table S5 –** Search strategy used in Lilacs via BVS

| Severe Obesity | Search in titles, abstracts and subject | "morbid obes*" or "severe obes*" or "obesity class iii" or "class iii obes*" or "severely obes*" or “obesidad mórbida” or “obesidad severa” or “obesidad clase iii” or “clase de obesidad iii” or “severamente obes*” or “obesidade mórbida” or “obesidade grave” or “obesidade severa” or “obesidade grau iii” |
| --- | --- | --- |
|  |  | **AND** |
| Quality of Life | Search in titles, abstracts and subject | "quality of life" or qol or hrqol or "life quality" or "health-related quality of life" or "health related quality of life" or “calidad de vida” or “qualidade de vida” or qv or qvrs or “qualidade de vida relacionada à saúde” |
|  |  | **AND NOT** |
| Limits for exclusion | Search in titles | "bariatric*" or "metabolic surgery" or "gastrectomy" or "roux-en-y" or "gastric bypass" or "gastric balloon" or “cirugía bariátrica” or “gastrectomía” or “bypass gástrico” or “cirugía metabólica” or “balón gástrico” or “bariátrica” or “cirurgia metabólica” or “gastrectomia” or “bypass gástrico” or “balão gástrico” |
| Resultados: 3 | | |

**Supplementary Table S6 –** Search strategy used in SciELO

| Severe Obesity | Searches in all indexes | (obesidade mórbida ) OR (obesidade grave) OR (morbid obes*) OR (severe obes*) OR (obesity class iii) OR (class iii obes*) OR (obesidade severa) OR (obesidade grau iii) |
| --- | --- | --- |
|  |  | **AND** |
| Quality of Life |  | (quality of life) OR (qol) OR (life quality) OR (qualidade de vida) OR (qvrs) |
|  |  | **AND** |
| Observational studies |  | (epidemiologic stud*) OR (cohort stud*) OR (cohort analys*) OR (cross-sectional) OR (observational stud*) OR (prevalence stud*) OR (incidence stud*) OR (followup) OR (follow-up) OR (longitudinal) OR (retrospective) OR (prospective) OR (transversal) OR (observacional) OR (epidemiológico) OR (prevalência) OR (coorte) OR (incidência) OR (prospectivo) OR (retrospectivo) |
|  |  | **AND NOT** |
| Limits for exclusion |  | (bariatric*) OR (metabolic surgery) OR (gastrectomy) OR (roux-en-y) OR (gastric bypass) OR (gastric balloon) OR (bariátrica) OR (cirurgia metabólica) OR (gastrectomia) OR (bypass gástrico) OR (balão gástrico) |
| Results: 25 | | |

**Supplementary Table S7 –** Search strategy used in CINAHL via EBSCO

| Severe Obesity | Full text searches | "morbid obes*" or "severe obes*" or "obesity class iii" or "class iii obes*" or "severely obes*" |
| --- | --- | --- |
|  |  | **AND** |
| Quality of Life | Searches in abstracts | "quality of life" or qol or hrqol or "life quality" or "health-related quality of life" or "health related quality of life" |
|  |  | **AND** |
| Observational studies | Searches in abstracts | "epidemiologic stud*" or "cohort stud*" or "cohort analys*" or "cross-sectional" or "observational stud*" or "prevalence stud*" or "incidence stud*" or "followup" or "follow-up" or "longitudinal" or "prospective" or "retrospective" |
|  |  | **AND NOT** |
| Limits for exclusion | Searches in titles | "bariatric*" or "metabolic surgery" or "gastrectomy" or "roux-en-y" or "gastric bypass" or "gastric balloon" |
| Results: 78 | | |

**Supplementary Table S8 –** Quality of Life results in studies using the EQ-5D instrument among individuals with severe obesity – Mean (SD)

| Study | Index Score | Mobility domain | Self-care domain | Activities domain | Pain domain | Anxiety domain | Visual Analogue Scale |
| --- | --- | --- | --- | --- | --- | --- | --- |
| TOTAL | | | | | | | |
| Busutil, 2017^a^ [32] | - | 44.7% | 23.6% | 32.9% | 47.2% | 33.5% | - |
| Luah, 2024 [39] | 0.79 (0.01) | - | - | - | - | - | - |
| Sach, 2007[45] | 0.62 (0.29) | - | - | - | - | - | 58.5 (15.9) |
| MALE | | | | | | | |
| Luah, 2023 [39] | 0.73 (0.01) | - | - | - | - | - | - |
| Søltoft, 2009 ^a^ [47] | - | 30.5% | 10.2% | 20.3% | 40.7% | 10.2% | - |
| FEMALE | | | | | | | |
| Luah, 2024 [39] | 0.69 (0.01) | - | - | - | - | - | - |
| Søltoft, 2009 ^a^ [47] | - | 35.4% | 9.8% | 22.6% | 48.0% | 30.6% | - |

Legend: EQ-5D – EuroQoL 5 dimension; SD – Standard deviation

^a^ Percentage of people with "some problems" or "extreme problems"

**Supplementary Table S9 –** Quality of Life results in studies using other instruments among individuals with severe obesity – Mean (SD)

| Study | SF-6D Total Score | IWQOL-lite Score | BRFSS Unhealthy days Physical | BRFSS Unhealthy days Mental | BRFSS Activity limitation days | BRFSS Unhealthy days Physical and mental | WHOQO  Total | WHOQOL - Physical | WHOQOL - Psychological | WHOQOL - Social | WHOQOL - Environment |
| --- | --- | --- | --- | --- | --- | --- | --- | --- | --- | --- | --- |
| TOTAL | | | | | | | | | | | |
| Ford, 2001 [35] | - | - | 6.5 (0.4) | 4.9 (0.3) | 3.7 (0.3) | 9.9 (0.4) | - | - | - | - | - |
| Kolotkin, 2002 [38] | - | 64.3 (22.9) | - | - | - | - | - | - | - | - | - |
| Sach, 2007 [45] | 0.7 (0.1) |  | - | - | - | - | - | - | - | - | - |
| MALE | | | | | | | | | | | |
| Pimenta, 2015 [42] |  |  | - | - | - | - | 56.1 (5.9) | 14.1 (1.3) | 13.2 (1.5) | 8.4 (1.1) | 20.6 (2.0) |
| FEMALE | | | | | | | | | | | |
| Pimenta, 2015 [42] |  |  | - | - | - | - | 58.1 (4.0) | 19.8 (2.4) | 12.6 (1.4) | 7.9 (0.7) | 21.9 (1.4) |

Legend: BRFSS HRQOL – CDC – Behavioral Risk Factor Surveillance System Health-Related Quality of Life – Centers for Disease; IWQOL-lite – Impact of Weight on Quality of Life-Lite; SD – Standard deviation; SF-6D – Short-Form 6 Dimensions; WHOQOL – World Health Organization Quality of Life Scale.

**Supplementary Table S10 –** Quality of Life results from studies using the SF-36 instrument by BMI categories.

|  |  | SF-36 Instrument Quality of Life Domains | | | | | | | | | |
| --- | --- | --- | --- | --- | --- | --- | --- | --- | --- | --- | --- |
| Study | **BMI categories** | **PCS** | **MCS** | **Physical function** | **Role physical** | **Bodily pain** | **General health** | **Mental health** | **Role emotional** | **Social function** | **Vitality** |
| MALE AND FEMALE | | | | | | | | | | | |
| Arbex, 2015 ^a^ [31] | NW | 83.4 (4.0) | - | - | - | - | - | - | - | - | - |
|  | OW | 50.1 (6.3) | - | - | - | - | - | - | - | - | - |
|  | OB.I/II | 54.9 (4.9) | - | - | - | - | - | - | - | - | - |
|  | OB.III | 44.2 (4.1) | - | - | - | - | - | - | - | - | - |
| Doll, 2000 ^a^[34] | UW | 50.6 (10.5) | 46.9 (10.9) | 87.8 (21.9) | 85.3 (24.1) | 80.1 (21.7) | 69.5 (22.5) | 68.3 (18.6) | 79.4 (24.3) | 77.5 (25.4) | 56.7 (20.3) |
|  | NW | 51.2 (9.17) | 50.1 (10.0) | 90.4 (17.7) | 88.9 (20.5) | 80.6 (22.0) | 73.3 (19.7) | 72.2 (18.0) | 86.4 (20.4) | 83.6 (22.7) | 59.2 (19.2) |
|  | OW | 49.4 (10.0) | 50.6 (9.48) | 87.1 (19.5) | 86.8 (21.7) | 78.1 (23.2) | 70.1 (20.2) | 72.8 (17.4) | 86.6 (20.0) | 83.7 (22.2) | 58.3 (19.3) |
|  | OB.I/II | 46.0 (12.1) | 49.2 (11.1) | 79.9 (24.5) | 80.8 (27.1) | 72.2 (26.0) | 64.2 (21.7) | 69.8 (20.2) | 83.1 (25.0) | 78.4 (27.0) | 52.6 (20.9) |
|  | OB.III | 40.6 (13.1) | 48.1 (10.9) | 65.7 (25.8) | 74.6 (26.8) | 64.9 (25.4) | 56.9 (24.2) | 66.3 (20.1) | 78.8 (26.0) | 72.1 (29.0) | 47.3 (22.7) |
| Knoesen, 2012 ^a^ [37] | OB.I | 60.0 (20.0) | 72.0 (21.3) | 78.9 (20.9) | 62.5 (44.6) | 33.5 (14.0) | 61.0 (22.1) | 74.3 (17.5) | 75.8 (37.0) | 78.7 (25.5) | 57.3 (22.9) |
|  | OB.II | 56.5 (16.4) | 65.1 (22.4) | 71.9 (17.5) | 65.8 (40.7) | 33.3 (13.8) | 54.8 (23.8) | 69.0 (22.8) | 71.0 (38.2) | 72.6 (24.0) | 47.9 (21.4) |
|  | OB.III | 45.1 (19.5) | 58.4 (21.7) | 55.3 (26.8) | 50.5 (40.3) | 26.5 (12.5) | 47.1 (12.7) | 65.7 (20.9) | 67.4 (39.4) | 60.9 (26.3) | 40.3 (19.4) |
| Palmeira, 2023 ^b^ [40] | OW | - | - | 75 (70-90) | 100 (50-100) | 61 (41-61) | 50 (47-67) | 76 (68-80) | 100 (100-100) | 87 (87-87) | 45 (30-70) |
|  | OB.I | - | - | 77 (52-93) | 100 (25-100) | 51 (41-72) | 57 (42-67) | 66 (56-76) | 66 (33-100) | 75 (50-87) | 52 (32-73) |
|  | OB.II | - | - | 80 (55-90) | 100 (87-100) | 61 (41-62) | 52 (42-67) | 64 (42-90) | 100 (16-100) | 75 (50-93) | 60 (50-80) |
|  | OB.III | - | - | 45 (25-80) | 25 (0-100) | 40 (20-61) | 48 (30-67) | 56 (40-80) | 33 (0-100) | 62 (50-75) | 50 (20-80) |
| Pazzianotto-Forti, 2017 [41] | NW | - | - | 95 (75–100)^b^ | 100 (100–100)^b^ | 84 (62–84)^b^ | 61.9 (14.3) **^a^** | 76 (70–84)^b^ | 100 (100–100)^b^ | 83.9 (29.7)**^a^** | 75 (72–80)^b^ |
|  | OW | - | - | 80 (70–100)^b^ | 100 (100–100)^b^ | 62 (62–62)^b^ | 57.0 (16.8) **^a^** | 82 (80–84)^b^ | 100 (100–100)^b^ | 90.3 (16.4)**^a^** | 80 (65–80)^b^ |
|  | OB | - | - | 70 (50–83)^b^ | 100 (50–100)^b^ | 51 (41–61)^b^ | 56.9 (14.8) **^a^** | 76 (68–80)^b^ | 100 (100–100)^b^ | 84.1 (18.9)**^a^** | 65 (52–79)^b^ |
|  | OB.III | - | - | 50 (35–67)^b^ | 50 (0–100)^b^ | 51 (30–62)^b^ | 53.7 (15.1)**^b^** | 68 (44–80)^b^ | 66 (33–100)^b^ | 69.3 (28.9)**^a^** | 60 (35–70)^b^ |
| Rozjabek, 2020 ^c^ [44] | NW | 52.4 | 46.9 | 51.6 | 49.9 | 51.0 | 51.7 | 47.6 | 47.4 | 48.1 | 50.6 |
|  | OW | 50.9 | 48.5 | 50.8 | 49.8 | 50.0 | 50.7 | 49.0 | 48.4 | 49.0 | 50.5 |
|  | OB.I | 48.4 | 47.5 | 48.5 | 48.0 | 47.8 | 47.8 | 47.9 | 47.1 | 47.6 | 48.3 |
|  | OB.II | 46.6 | 45.7 | 46.5 | 46.2 | 46.4 | 45.4 | 46.3 | 45.0 | 45.7 | 46.2 |
|  | OB.III | 43.0 | 43.2 | 42.5 | 43.1 | 43.4 | 42.0 | 43.6 | 42.0 | 42.7 | 43.1 |
| Yancy, 2002 [48] | NW | 37.1 (12.3) | 49.9 (13.5) | 56.7 (30.2) | 50.8 (42.9) | 53.1 (27.4) | 52.4 (25.4) | 72.5 (24.4) | 68.5 (42.2) | 70.4 (29.6) | 50.0 (26.9) |
|  | OW | 37.0 (12.4) | 49.6 (14.2) | 58.2 (30.2) | 50.6 (43.5) | 48.3 (26.3) | 53.5 (24.9) | 71.2 (25.2) | 69.9 (41.4) | 69.8 (30.7) | 49.4 (26.4) |
|  | OB.I | 35.4 (11.9) | 48.8 (14.4) | 52.1 (30.3) | 44.0 (42.6) | 46.8 (26.2) | 52.8 (24.1) | 69.1 (26.1) | 64.2 (41.7) | 69.1 (31.1) | 47.0 (26.5) |
|  | OB.II | 33.6 (10.9) | 51.9 (13.2) | 50.1 (27.4) | 43.9 (41.6) | 45.4 (23.9) | 51.7 (23.4) | 73.8 (23.7) | 74.5 (38.5) | 68.5 (32.4) | 49.2 (25.6) |
|  | OB.III | 28.5 (0.2) | 48.8 (15.4) | 35.0 (23.1) | 27.5 (37.3) | 38.8 (25.1) | 47.2 (23.6) | 67.5 (28.5) | 68.9 (42.8) | 57.9 (29.8) | 33.0 (27.1) |
| MALE | | | | | | | | | | | |
| Hopman, 2007 [36] | UW | 46.3 (9.80) | 51.2 (9.80) | 71.6 (28.5) | 75.0 (34.5) | 74.7 (24.3) | 69.7 (21.3) | 77.0 (19.3) | 81.8 (33.7) | 75.0 (27.3) | 61.4 (18.7) |
|  | NW | 49.2 (9.28) | 53.4 (8.38) | 82.0 (22.2) | 80.5 (34.2) | 77.4 (22.5) | 74.4 (18.2) | 80.1 (14.2) | 84.9 (30.3) | 87.8 (19. 9) | 68.0 (17.5) |
|  | OW | 49.4 (9.19) | 54.5 (7.72) | 82.7 (21.5) | 83.1 (32.1) | 77.7 (22.2) | 75.9 (17.6) | 81.8 (13.7) | 89.3 (26.9) | 89.7 (18.1) | 68.8 (17.7) |
|  | OB.I | 47.8 (9.6) | 54.3 (7.9) | 79.3 (21.8) | 80.7 (33.7) | 75.1 (22.6) | 72.6 (18.2) | 81.2 (13.9) | 88.3 (26.9) | 88.4 (19.2) | 66.3 (17.8) |
|  | OB.II | 46.72 (9.6) | 53.8 (9.0) | 74.8 (23.5) | 76.5 (37.1) | 74.6 (25.5) | 71.2 (17.8) | 80.5 (16.1) | 82.9 (33.7) | 87.5 (20.8) | 64.9 (20.3) |
|  | OB.III | 40.55 (10.0) | 56.0 (6.3) | 63.0 (27.4) | 62.5 (41.7) | 62.8 (24.2) | 66.3 (19.0) | 84.0 (9.3) | 81.7 (35.0) | 80.0 (21.2) | 65.3 (16.4) |
| Renzaho, 2010 ^a^ [43] | UW | 45.5 (14.1) | 46.0 (12.3) | 64.9 (32.6) | 54.7 (48.2) | 56.3 (31.3) | 59.0 (26.4) | 60.4 (28.1) | 54.4 (45.2) | 58.5 (35.5) | 44.7 (25.1) |
|  | NW | 52.0 (9.5) | 50.3 (8.8) | 86.4 (22.4) | 81.7 (34.5) | 75.9 (22.8) | 70.4 (20.5) | 75.3 (15.5) | 85.5 (30.6) | 84.3 (22.1) | 63.7 (18.7) |
|  | OW | 50.7 (9.7) | 50.8 (9.1) | 84.3 (22.0) | 80.0 (35.7) | 73.4 (23.4) | 67.8 (20.6) | 76.1 (16.5) | 84.5 (31.9) | 84.7 (22.3) | 62.5 (18.9) |
|  | OB.I | 49.5 (9.6) | 50.6 (9.4) | 80.8 (23.5) | 77.8 (36.2) | 71.4 (23.7) | 65.0 (20.6) | 74.6 (17.6) | 84.0 (32.8) | 83.6 (22.4) | 60.7 (18.9) |
|  | OB.II | 48.4 (10.1) | 49.9 (9.8) | 79.0 (24.1) | 71.5 (39.5) | 70.8 (25.4) | 60.1 (20.6) | 73.3 (18.0) | 82.6 (33.9) | 80.7 (24.5) | 57.4 (19.3) |
|  | OB.III | 42.9 (12.3) | 49.9 (11.5) | 66.3 (30.4) | 61.0 (44.1) | 59.2 (30.6) | 51.2 (23.1) | 70.9 (21.6) | 73.8 (41.7) | 74.1 (29.6) | 57.7 (23.7) |
| Slagter, 2015 ^d^ [46] | NW | - | - | 21.9% | 16.1% | 23.1% | 20.1% | 21.7% | 10.9% | 19.8% | 18.8% |
|  | OB.I No DM2 | - | - | 46.6% | 24.1% | 33.4% | 31.8% | 21.9% | 13.0% | 23.4% | 24.7% |
|  | OB.I DM2 | - | - | 63.3% | 28.8% | 39.6% | 43.2% | 26.9% | 16.8% | 31.3% | 29.6% |
|  | OB.II No DM2 | - | - | 60.9% | 29.0% | 39.6% | 41.5% | 27.1% | 15.4% | 29.6% | 34.9% |
|  | OB.II DM2 | - | - | 75.6% | 37.9% | 48.3% | 55.3% | 35.8% | 18.2% | 32.8% | 39.6% |
|  | OB.III No DM2 | - | - | 72.1% | 28.3% | 38.4% | 46.1% | 28.5% | 17.4% | 34.4% | 39.0% |
|  | OB.III DM2 | - | - | 91.7% | 38.7% | 38.9% | 61.0% | 22.2% | 22.3% | 30.3% | 41.5% |
| FEMALE | | | | | | | | | | | |
| Hopman, 2007 ^a^ [36] | UW | 43.8 (12.6) | 51.5 (10.9) | 63.7 (32.0) | 68.6 (41.9) | 69.7 (27.1) | 67.7 (22.8) | 74.0 (19.0) | 78.2 (37.8) | 80.8 (26.0) | 58.5 (24.9) |
|  | NW | 48.1 (10.0) | 53.1 (8.6) | 78.6 (23.2) | 77.3 (36.5) | 73.8 (23.7) | 76.1 (18.1) | 78.3 (15.0) | 85.4 (30.5) | 86.8 (20.0) | 64.9 (18.7) |
|  | OW | 47.0 (10.0) | 52.9 (9.0) | 75.5 (23.3) | 75.3 (37.4) | 71.6 (24.2) | 74.4 (18.2) | 77.8 (15.4) | 83.8 (31.9) | 86.2 (20.7) | 63.3 (19.5) |
|  | OB.I | 44.9 (10.5) | 53.1 (9.4) | 68.8 (25.2) | 72.3 (38.9) | 68.6 (24.5) | 72.0 (18.9) | 77.7 (15.8) | 82.0 (33.4) | 84.2 (22.0) | 60.8 (20.0) |
|  | OB.II | 42.2 (11.7) | 53.4 (9.0) | 63.3 (27.0) | 66.0 (41.3) | 62.8 (26.3) | 69.8 (20.4) | 77.7 (15.9) | 80.2 (33.9) | 81.7 (24.1) | 58.2 (21.9) |
|  | OB.III | 38.3 (12.5) | 52.0 (9.9) | 55.3 (28.4) | 53.9 (42.3) | 57.7 (25.9) | 60.2 (24.5) | 75.4 (17.7) | 72.1 (39.6) | 76.0 (23.7) | 53.8 (20.7) |
| Renzaho, 2010 ^a^ [43] | UW | 49.2 (11.0) | 46.0 (11.5) | 77.7 (26.4) | 68.9 (41.2) | 71.0 (28.3) | 60.7 (25.5) | 67.3 (19.5) | 74.7 (40.0) | 73.2 (30.1) | 52.7 (25.6) |
|  | NW | 52.1 (9.3) | 49.4 (9.5) | 85.5 (21.3) | 81.4 (33.9) | 75.2 (23.1) | 71.9 (20.4) | 73.8 (16.9) | 84.5 (31.5) | 83.1 (22.7) | 60.9 (19.4) |
|  | OW | 49.5 (10.7) | 49.8 (10.0) | 78.9 (24.6) | 75.0 (38.6) | 69.7 (26.1) | 67.8 (22.0) | 73.5 (17.5) | 82.0 (34.0) | 81.6 (23.7) | 58.5 (19.9) |
|  | OB.I | 46.5 (11.6) | 49.4 (10.0) | 73.7 (25.7) | 68.3 (41.3) | 64.5 (25.6) | 61.7 (22.0) | 72.4 (17.1) | 79.8 (34.9) | 76.7 (25.6) | 54.4 (20.0) |
|  | OB.II | 45.6 (10.7) | 50.0 (9.9) | 71.3 (26.0) | 67.5 (41.3) | 62.5 (25.1) | 59.6 (23.0) | 72.4 (18.7) | 77.9 (36.8) | 77.6 (26.2) | 54.0 (20.7) |
|  | OB.III | 42.6 (11.6) | 47.9 (11.3) | 63.1 (26.6) | 60.1 (43.4) | 56.4 (27.3) | 53.1 (23.2) | 67.6 (19.5) | 72.5 (40.5) | 71.1 (26.9) | 47.7 (22.3) |
| Slagter, 2015^d^ [46] | NW | - | - | 20.4% | 20.6% | 12.2% | 20.9% | 23.2% | 14.8% | 17.4% | 19.7% |
|  | OB.I No DM2 | - | - | 43.9% | 29.0% | 20.5% | 32.0% | 24.7% | 17.6% | 21.3% | 26.4% |
|  | OB.I DM2 | - | - | 63.5% | 38.8% | 28.0% | 47.2% | 28.7% | 21.1% | 23.7% | 31.4% |
|  | OB.II No DM2 | - | - | 54.4% | 33.5% | 34.8% | 40.1% | 26.6% | 19.3% | 23.8% | 30.2% |
|  | OB.II DM2 | - | - | 66.4% | 38.5% | 34.8% | 54.1% | 29.5% | 21.1% | 30.0% | 32.1% |
|  | OB.III No DM2 | - | - | 69.2% | 41.0% | 32.9% | 53.4% | 31.7% | 24.2% | 28.7% | 34.3% |
|  | OB.III DM2 | - | - | 83.0% | 52.3% | 42.0% | 67.0% | 36.2% | 27.3% | 31.7% | 43.3% |

^a^ Mean (standard deviation)

^b^ Median (interquartile range)

^c^ Mean

^d^ Percentage (%) of individuals with low scores in each quality of life domains

Legend: DM2 – Diabetes mellitus; MCS – Mental Component Score; NW – Normal weight; OB.I – Obesity class I; OB.II – Obesity class II; OB.III – Obesity class III; OW – Overweight; PCS – Physical Component Score; SF-36 – 36-Item Short Form Health Survey; UW – Underweight.

**Supplementary Table S11 –** Quality of Life results from studies using the EQ-5D instrument by BMI categories

|  |  | EQ-5D Instrument Quality of Life Domains | | | | | | |
| --- | --- | --- | --- | --- | --- | --- | --- | --- |
| Study | BMI categories | Index Score | Mobility domain | Self-care domain | Activities domain | Pain domain | Anxiety domain | Visual Analogue Scale |
| TOTAL | | | | | | | | |
| Busutil, 2017^a^ [32] | NW | - | 9.4% | 4.3% | 7.9% | 20.3% | 13.0% | - |
|  | OB.I | - | 24.8% | 10.4% | 18.6% | 36.8% | 21.0% | - |
|  | OB.II | - | 37.2% | 15.8% | 27.4% | 48.0% | 27.4% | - |
|  | OB.III | - | 44.7% | 23.6% | 32.9% | 47.2% | 33.5% | - |
| Luah, 2024 ^b^ [39] | UW | 0.85 (0.20) | - | - | - | - | - | - |
|  | NW | 0.87 (0.00) | - | - | - | - | - | - |
|  | OW | 0.86 (0.00) | - | - | - | - | - | - |
|  | OB.I | 0.82 (0.00) | - | - | - | - | - | - |
|  | OB.II | 0.79 (0.01) | - | - | - | - | - | - |
|  | OB.III | 0.79 (0.01) | - | - | - | - | - | - |
| Sach, 2007 ^b^ [45] | UW | 0.76 (0.17) | - | - | - | - | - | 75.1 (17.8) |
|  | NW | 0.80 (0.22) | - | - | - | - | - | 77.8 (16.0) |
|  | OW | 0.78 (0.24) | - | - | - | - | - | 75.4 (17.4) |
|  | OB.I | 0.70 (0.29) | - | - | - | - | - | 69.8 (18.8) |
|  | OB.II | 0.68 (0.24) | - | - | - | - | - | 66.8 (17.3) |
|  | OB.III | 0.62 (0.29) | - | - | - | - | - | 58.5 (15.9) |
| MALE | | | | | | | | |
| Luah, 2023 ^b^ [39] | UW | 0.86 (0.03) | - | - | - | - | - | - |
|  | NW | 0.88 (0.00) | - | - | - | - | - | - |
|  | OW | 0.87 (0.00) | - | - | - | - | - | - |
|  | OB.I | 0.84 (0.01) | - | - | - | - | - | - |
|  | OB.II | 0.83 (0.01) | - | - | - | - | - | - |
|  | OB.III | 0.73 (0.01) | - | - | - | - | - | - |
| Søltoft, 2009 ^a^ [47] | UW | - | 11.3% | 7.6% | 20.8% | 28.3% | 28.3% | - |
|  | NW | - | 11.1% | 3.0% | 11.7% | 24.4% | 15.6% | - |
|  | OW | - | 14.6% | 3.3% | 12.2% | 27.3% | 14.5% | - |
|  | OB.I/II | - | 21.8% | 4.5% | 18.3% | 36.9% | 16.6% | - |
|  | OB.III | - | 30.5% | 10.2% | 20.3% | 40.7% | 10.2% | - |
| FEMALE | | | | | | | | |
| Luah, 2024 ^b^ [39] | UW | 0.84 (0.02) | - | - | - | - | - | - |
|  | NW | 0.87 (0.00) | - | - | - | - | - | - |
|  | OW | 0.84 (0.00) | - | - | - | - | - | - |
|  | OB.I | 0.80 (0.01) | - | - | - | - | - | - |
|  | OB.II | 0.77 (0.01) | - | - | - | - | - | - |
|  | OB.III | 0.69 (0.01) | - | - | - | - | - | - |
| Søltoft, 2009 ^a^ [47] | UW | - | 20.6% | 7.6% | 15.9% | 25.5% | 31.1% | - |
|  | NW | - | 10.1% | 2.6% | 11.2% | 25.4% | 21.3% | - |
|  | OW | - | 17.2% | 3.5% | 15.1% | 35.2% | 20.4% | - |
|  | OB.I/II | - | 28.0% | 5.7% | 22.0% | 44.4% | 23.8% | - |
|  | OB.III | - | 35.4% | 9.8% | 22.6% | 48.0% | 30.6% | - |

^a^ Percentage of people with "some problems" or "extreme problems"

^b^ Mean (standard deviation)

Legend: NW – Normal weight; OB.I – Obesity class I; OB.II – Obesity class II; OB.III – Obesity class III; OW – Overweight; UW – Underweight.

**Supplementary Table S12 –** Quality of Life results from studies using others instruments by BMI categories

|  |  | Instruments and their Quality of Life Domains - Mean (SD) | | | | | | | | | | |
| --- | --- | --- | --- | --- | --- | --- | --- | --- | --- | --- | --- | --- |
|  |  | **SF-6D** | **IWQOL-Lite** | **BRFSS-CDC** | | | | **WHOQOL-Bref** | | | | |
| Study | **BMI Category** | **Total Score** | **Total Score** | **Unhealthy days Physical** | **Unhealthy days Mental** | **Activity limitation days** | **Unhealthy days Physical and mental** | **Total Score** | **Physical** | **Psychological** | **Social** | **Environment** |
| MALE AND FEMALE | | | | | | | | | | | | |
| Ford, 2001 [35] | UW | - | - | 4.0 (0.2) | 4.0 (0.2) | 2.6 (0.2) | 7.0 (0.3) | - | - | - | - | - |
|  | NW | - | - | 2.5 (0.0) | 2.8 (0.0) | 1.4 (0.0) | 4.8 (0.1) | - | - | - | - | - |
|  | OW | - | - | 2.7 (0.1) | 2.5. (0.1) | 1.5 (0.0) | 4.7 (0.1) | - | - | - | - | - |
|  | OB.I | - | - | 3.8 (0.1) | 3.3 (0.1) | 2.2 (0.1) | 6.2 (0.1) | - | - | - | - | - |
|  | OB.II | - | - | 5.3 (0.3) | 4.4 (0.2) | 3.5 (0.2) | 8.4 (0.3) | - | - | - | - | - |
|  | OB.III | - | - | 6.5 (0.4) | 4.9 (0.3) | 3.7 (0.3) | 9.9 (0.4) | - | - | - | - | - |
| Kolotkin, 2002 [38] | OW | - | 93.6 (8.2) | - | - | - | - | - | - | - | - | - |
|  | OB.I | - | 89.1 (11.1) | - | - | - | - | - | - | - | - | - |
|  | OB.II | - | 81.2 (16.4) | - | - | - | - | - | - | - | - | - |
|  | OB.III | - | 64.3 (22.9) | - | - | - | - | - | - | - | - | - |
| Sach, 2007 [45] | UW | 0.7 (0.1) | - | - | - | - | - | - | - | - | - | - |
|  | NW | 0.8 (0.1) | - | - | - | - | - | - | - | - | - | - |
|  | OW | 0.8 (0.1) | - | - | - | - | - | - | - | - | - | - |
|  | OB.I | 0.7 (0.2) | - | - | - | - | - | - | - | - | - | - |
|  | OB.II | 0.7 (0.1) | - | - | - | - | - | - | - | - | - | - |
|  | OB.III | 0.7 (0.1) | - | - | - | - | - | - | - | - | - | - |
| MALE | | | | | | | | | | | | |
| Pimenta, 2015 [42] | NW | - | - | - | - | - | - | 89.2 (4.9) | 27.2 (1.8) | 21.7 (1.7) | 11.0 (0.6) | 29.2 (1.2) |
|  | OB | - | - | - | - | - | - | 79.5 (3.6) | 23.0 (1.3) | 18.7 (1.1) | 10.7 (0.6) | 26.7 (1.2) |
|  | OB.III | - | - | - | - | - | - | 56.1 (5.9) | 14.1 (1.3) | 13.2 (1.5) | 8.4 (1.1) | 20.6 (2.0) |
| FEMALE | | | | | | | | | | | | |
| Pimenta, 2015 [42] | NW | - | - | - | - | - | - | 91.3 (2.3) | 28.2 (1.0) | 23.3 (1.1) | 12.0 (0.5) | 27.4 (1.0) |
|  | OB | - | - | - | - | - | - | 67.8 (6.8) | 19.8 (2.4) | 16.5 (1.9) | 9.8 (0.7) | 21.5 (2.2) |
|  | OB.III | - | - | - | - | - | - | 58.1 (4.0) | 19.8 (2.4) | 12.6 (1.4) | 7.9 (0.7) | 21.9 (1.4) |

Legend: BRFSS HRQOL – CDC – Behavioral Risk Factor Surveillance System Health-Related Quality of Life – Centers for Disease; IWQOL-lite – Impact of Weight on Quality of Life-Lite; NW – Normal weight; OB.I – Obesity class I; OB.II – Obesity class II; OB.III – Obesity class III; OW – Overweight; SD – Standard deviation;SF-6D – Short-Form 6 Dimensions; UW – Underweight; WHOQOL-Bref – World Health Organization Quality of Life Scale.

**Supplementary Figure S1** – Meta-analysis of the SF-36 Physical Function domain

Legend: REML – restricted maximum likelihood model; SF-36 – 36-Item Short Form Health Survey.

**Supplementary Figure S2** – Meta-analysis of the SF-36 Role Physical domain

Legend: REML – restricted maximum likelihood model; SF-36 – 36-Item Short Form Health Survey.

**Supplementary Figure S3 –** Meta-analysis of the SF-36 Bodily Pain domain

Legend: REML – restricted maximum likelihood model; SF-36 – 36-Item Short Form Health Survey.

**Supplementary Figure S4 –** Meta-analysis of the SF-36 General Health domain

Legend: REML – restricted maximum likelihood model; SF-36 – 36-Item Short Form Health Survey.

**Supplementary Figure S5 –** Meta-analysis of the SF-36 Mental Health domain

Legend: REML – restricted maximum likelihood model; SF-36 – 36-Item Short Form Health Survey.

**Supplementary Figure S6 –** Meta-analysis of the SF-36 Role Emotional domain

Legend: REML – restricted maximum likelihood model; SF-36 – 36-Item Short Form Health Survey.

**Supplementary Figure S7 –** Meta-analysis of the SF-36 Social Function domain

Legend: REML – restricted maximum likelihood model; SF-36 – 36-Item Short Form Health Survey.

**Supplementary Figure S8 –** Meta-analysis of the SF-36 Vitality domain

Legend: REML – restricted maximum likelihood model; SF-36 – 36-Item Short Form Health Survey.
